# Neoantigen-Reactive T Cells: The Driving Force behind Successful Melanoma Immunotherapy

**DOI:** 10.3390/cancers13236061

**Published:** 2021-12-01

**Authors:** Lindy Davis, Ashley Tarduno, Yong-Chen Lu

**Affiliations:** 1 Department of Surgery, Albany Medical Center, Albany, NY 12208, USA; davisl8@amc.edu (L.D.); tarduna@amc.edu (A.T.); 2 Department of Pathology, University of Arkansas for Medical Sciences, Little Rock, AR 72205, USA; 3 Winthrop P. Rockefeller Cancer Institute, University of Arkansas for Medical Sciences, Little Rock, AR 72205, USA

**Keywords:** immunotherapy, neoantigen, T cell, melanoma

## Abstract

**Simple Summary:**

Cancer immunotherapy is a revolutionary type of cancer therapy. It uses the patient’s own immune system to fight and potentially cure cancer. The first major breakthrough of immunotherapy came from successful clinical trials for melanoma treatments. Since then, researchers have focused on understanding the science behind immunotherapy, so that patients with other types of cancer may also benefit. One of the major findings is that the T cells in melanoma patients may recognize a specific type of tumor antigen, called neoantigens, and then kill tumor cells that present these neoantigens. The neoantigens mainly arise from the DNA mutations found in tumor cells. These mutations are translated into mutated proteins that are then distinguished by T cells. In this article, we discuss the critical role of T cells in immunotherapy, as well as the clinical trials that shaped the treatments for melanoma.

**Abstract:**

Patients with metastatic cutaneous melanoma have experienced significant clinical responses after checkpoint blockade immunotherapy or adoptive cell therapy. Neoantigens are mutated proteins that arise from tumor-specific mutations. It is hypothesized that the neoantigen recognition by T cells is the critical step for T-cell-mediated anti-tumor responses and subsequent tumor regressions. In addition to describing neoantigens, we review the sentinel and ongoing clinical trials that are helping to shape the current treatments for patients with cutaneous melanoma. We also present the existing evidence that establishes the correlations between neoantigen-reactive T cells and clinical responses in melanoma immunotherapy.

## 1. Cutaneous Melanoma and the Immune System

Because tumor cells arise from the self, it is difficult for the immune system to distinguish between tumor and normal cells. Research in the past four decades has shed light on how the immune system recognizes and kills tumor cells. Here, we describe cutaneous melanoma and the immune system, focusing on the ways in which neoantigens may provide a unique opportunity for the immune system to recognize tumor cells.

### 1.1. The Tumorigenesis of Cutaneous Melanoma

Cutaneous melanoma is the most common type of melanoma [1]. It arises from melanocytes, a small population of cells within the skin. Melanocytes are highly specialized in the production of melanin—which is called melanogenesis—and thus baseline skin pigmentation. Melanocytes may undergo a transformation and become malignant. This occurs due to intrinsic genetic predisposition, hormonal regulation and environmental ultraviolet (UV) exposure [2,3]. Importantly, melanogenesis and melanogenesis-associated signaling pathways may have a strong influence in the tumorigenesis of melanoma, as well as therapeutic outcomes [4,5,6,7]. Additionally, the process of melanogenesis generates reactive oxygen species, quinone and semiquinone intermediates, which may create an immunosuppressive tumor microenvironment [8]. On the other hand, proteins associated with the melanin production are often highly expressed in melanoma, and they have become specific targets for molecular diagnostics and treatments, including immunotherapy (Section 1.5.1) [9].

### 1.2. The Immune System against Cancer

Immune cells distinguish the difference between the self and non-self, and subsequently attack foreign pathogens. The immune system in humans contains both innate and adaptive mechanisms. The innate immune system uses imprinted components, and is ready to recognize foreign pathogens without any previous encounter with these pathogens. Natural killer cells are an important cell type in the innate immunity against cancer as they are activated and kill target cells based on the lack of major histocompatibility complex (MHC) molecules [10]. Although MHC molecules are expressed in almost all normal cells, a subset of tumor cells may reduce or abolish the expression of MHC molecules, leading to the activation of NK cells. However, no strong evidence has been provided yet to demonstrate that NK cells play a major role in anti-tumor immunity in humans. An interesting recent development is chimeric antigen receptor (CAR)-NK cell therapy. NK cells were genetically modified with CAR to re-direct the specificity of NK cells to kill CD19^+^ B cell leukemia or lymphoma [11]. In a phase I/II study, complete responses were observed in seven out of 11 patients with non-Hodgkin’s lymphoma or chronic lymphocytic leukemia. Another important component of the innate immunity is antigen-presenting cells, including macrophages and dendritic cells [12]. In addition to presenting antigens expressed by tumor cells, these cells may also sense danger by recognizing unique molecular patterns generated by tumor cells [13,14]. For instance, high-mobility group box 1 (HMGB1) can be secreted from dying tumor cells and detected by toll-like receptors (TLRs) and advanced glycation end products (RAGE) on dendritic cells and macrophages, leading to downstream immune responses [15]. Cancer immunotherapies targeting innate immune signaling are currently under intensive study [16].

Adaptive immune responses are driven by T cells and B cells. The specificity and diversity of these cells form the basis for the adaptive immunity against pathogens and cancer. The primary function of B cells is the production of antigen-specific antibodies against pathogens, and anti-tumor antibodies are found in the serum and the tumor microenvironment in cancer patients [17,18]. Recent studies suggest that tumor-infiltrating B cells might possess certain functions in the tumor microenvironment, indicated by unique gene signatures [19,20]. CD20^+^ B cells and tertiary lymphoid structures were strongly associated with preferred clinical outcomes for melanoma patients treated with checkpoint blockade immunotherapy. The enriched memory B cells may help to promote the levels of TCF1^+^ memory T cells, which have been proposed as one the critical cell populations for successful checkpoint immunotherapy (Section 5.5) [21,22,23,24,25].

CD8^+^ cytotoxic T cells are thought to play a major role in immune responses and immunotherapies against cancer. Numerous examples may be found in the upcoming sections. CD4^+^ helper T cells were thought to provide help for CD8^+^ T cells, but accumulated evidence supports the hypothesis that CD4^+^ T cells may play a central and indispensable role in anti-tumor immunity [26,27]. Without the help of CD8^+^ T cells, transferring tumor antigen-specific CD4^+^ T cells induced tumor regressions in both mice and humans [28,29]. Importantly, the loss of β-2 microglobulin (B2M) has been identified as one of the dominant mechanisms for tumor cells to escape immunotherapy. B2M is an essential component of MHC class I, which mediates antigen presentation for CD8^+^ T cells. The escape mechanism may be overcome by utilizing CD4^+^ T cells, the activation of which does not depend on B2M [30].

### 1.3. Antigen Process and Presenting Pathways

T cells may detect non-self, short peptides, also known as T-cell epitopes, presented by MHC molecules on the cell surface [12]. These short peptides are originally derived from endogenous proteins made by transcriptional and translational processes. Some of these proteins are processed into short peptides by proteasomes. In antigen-presenting cells, these proteins may also come from extracellular sources and be processed differently by another type of proteasomes, called immunoproteasomes, resulting in different short peptides [31,32]. A small portion of the short peptides are able to bind to MHC molecules, based on their affinities to the specific types of MHC molecules. In general, short peptides 8–11 amino acids in length may bind to MHC class I molecules. In contrast, short peptides 15–18 amino acids in length may bind to MHC class II molecules. Due to the structure of MHC class II molecules, slightly longer peptides are also able to bind to MHC class II molecules. Another important structural difference is that MHC class II molecules have an α and a β chain, and the correct pairing is important for their function and specificity [33]. On the other hand, MHC class I molecules only have a single chain pairing with the shared B2M molecule. The loss of B2M impairs the function of the MHC class I molecule [34,35]. Lastly, peptide/MHC class I and II complexes traffic to the cell surface, where T-cell receptors (TCR) expressed on CD8^+^ and CD4^+^ T cells, respectively, may interact and potentially recognize these short peptides.

### 1.4. Co-Inhibitory Molecules

The interactions between TCRs and MHC molecules serve as the on/off “switch” for the interactions between T cells and tumor cells, or between T cells and antigen-presenting cells. In addition to this switch, other molecules, also known as co-stimulatory or co-inhibitory molecules, serve as fine tuners and modulate the T-cell responses. In general, co-stimulatory molecules enhance T-cell responses, while co-inhibitory molecules serve as “checkpoints” to prevent T cells from becoming over-reactive by providing inhibitory signals.

CTLA-4 (cytotoxic T-lymphocyte-associated protein 4) was the first dominant T-cell co-inhibitory molecule that was identified. A large number of self-reactive T cells were found in the peripheral organs of mice without the CTLA-4 co-inhibitory molecule [36,37]. Subsequently, the blockade of CTLA-4 could re-activate the T cells, and CTLA-4 blockade antibody could induce tumor rejection in mice [38]. This pioneer study serves as the foundation for checkpoint blockade immunotherapy.

PD-1 (programmed death 1) is another important co-inhibitory molecule, which was originally identified as a molecule induced upon programmed cell death [39]. Later studies showed that PD-1 serves as a co-inhibitory molecule in T cells, but the phenotype of PD-1-deficient mice is not as strong as that of CTLA-4-deficient mice [40,41]. Interestingly, the clinical activity of the PD-1 blockade antibody was far superior to the CTLA-4 blockade antibody in clinical trials (Section 4). With the success of PD-1 checkpoint blockade immunotherapy, many other co-inhibitory molecules, such as LAG-3 (lymphocyte-activation gene 3), have been actively studied in clinical trials [42].

### 1.5. Overview of Tumor Antigens

There are four major types of tumor antigens that may be recognized by T cells, including tissue-specific differentiation antigens, cancer germline antigens, viral antigens and neoantigens.

#### 1.5.1. Tissue-Specific Differentiation Antigens

A subset of T cells may pass through the negative selection process in the thymus, and they may recognize some normal proteins that are highly expressed in specific cell types or tissues [43]. For instance, normal melanocytes and melanoma cells almost exclusively express several proteins at high levels, and these proteins may be recognized by T cells after being processed and presented on the cell surface. These antigens, including MART-1, gp100 and tyrosinase, are named as melanocyte differentiation antigens [44,45]. Because normal melanocytes also express these antigens, highly activated T cells may attack these normal cells, in addition to tumor cells, leading to potentially severe toxicities [46].

#### 1.5.2. Cancer Germline Antigens

Cancer germline antigens are a class of antigens which are almost exclusively expressed in normal germline cells and some types of tumor cells. Male germ cells and trophoblastic cells lack the expression of MHC class I molecules to present antigens; thus, T cells specific to cancer germline antigens cannot recognize and attack germ cells. As a result, cancer germline antigens become ideal targets for T cell-based immunotherapies. The first cancer germline antigen, named melanoma antigen-1 (MAGE-A1), was discovered in 1991 [47,48]. In addition to testis, MAGE-A1 was highly expressed in about 50% of melanoma specimens and approximately 10 to 50% of several other types of cancer [49,50]. After the discovery of MAGE-A1, dozens of cancer germline antigens were discovered, and they have been studied intensively as targets of immunotherapy.

#### 1.5.3. Viral Antigens

It is well-known that several types of viruses may induce carcinogenesis in humans, and they are obviously ideal targets for cancer prevention and immunotherapy [51]. For example, human papillomaviruses (HPVs) may induce cervical cancer, and head and neck cancer [52]. HPV vaccines have been used globally to prevent cervical cancer, and HPV oncoproteins E6 and E7 have been targeted for cancer immunotherapies, such as adoptive cell therapy (Section 5.3). Autologous T cells were genetically modified to target HPV E6 or E7 and then adoptively transferred back to patients to treat HPC-associated epithelial cancers. In the initial phase I/II clinical trial, the targeting of HPV16 E6 resulted in two objective clinical responses in 12 patients [53], while the targeting of HPV16 E7 resulted in six objective clinical in 12 patients [54]. Although viral antigens are attractive targets for cancer immunotherapies, they are limited to virus-induced cancers.

#### 1.5.4. Neoantigens

**Figure 1:**
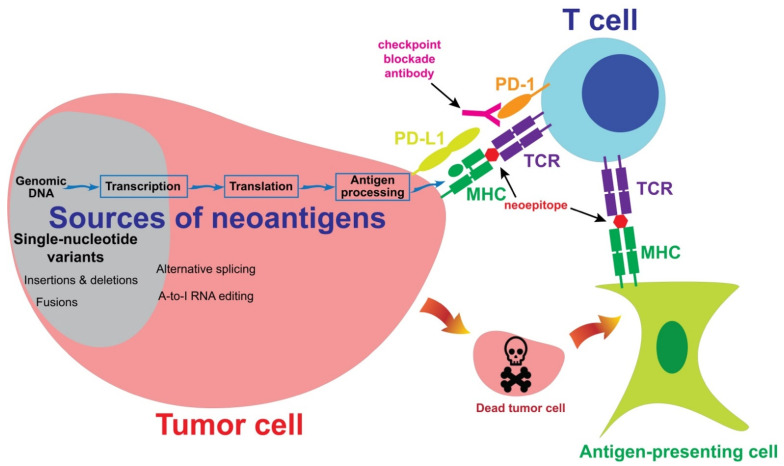
The sources of neoantigens. In cutaneous melanoma, the majority of neoantigens are generated from single-nucleotide variants. Other sources of neoantigens include insertions, deletions, fusions and alternative splicing. A subset of T cells may recognize neoepitopes presented by tumor cells or antigen-presenting cells. Neoantigen-reactive T cells then kill tumor cells in an antigen-specific manner. Co-inhibitory molecules, such as PD-1, may inhibit T cell-mediated immune responses against tumor cells. A checkpoint blockade antibody may block the inhibitory signaling and enhance immune responses.

Neoantigens are mutated proteins that arise from tumor-specific mutations. The current evidence supports the hypothesis that neoantigens may provide opportunities for T cells to recognize tumor cells [55,56]. Figure 1 summarizes the interaction between a neoantigen-reactive T cell and a tumor cell. Neoantigens may be generated from alternations in the genomic DNA, or in the post-transcriptional or post-translational steps. These neoantigens are then processed and presented on the tumor cell surface, after which the neoantigens may be recognized by a neoantigen-reactive T cell. Alternatively, an antigen-presenting cell may take neoantigens which were originally generated from a tumor cell and then present them to a neoantigen-reactive T cell. In the following sections, we will describe the sources of neoantigens and the approaches to the identification of these neoantigens.

## 2. Sources of Neoantigens

### 2.1. Single-Nucleotide Variants

The most dominant source of neoantigens in melanoma comes from single-nucleotide variants (Figure 1). The majority of melanomas are caused by UV light-induced DNA damage, which leads to cytidine to thymidine (C > T) transitions in genomic DNA [57,58]. After transcription and translation, some of these C > T mutations produce amino acid changes, also known as nonsynonymous mutations. One of the most interesting features of neoantigens generated from single-nucleotide variants is that a T-cell receptor (TCR) may differentiate a single amino acid change and react to the mutated epitope, but not the wild-type epitope. In some rare cases, TCRs do not have sufficient specificities, recognizing both wild-type and mutated epitopes. In such cases, these single-nucleotide variants should not be considered neoantigens.

Mutated CDK4 (R24C) and CTNNB1 (S37F) were among the first melanoma neoantigens to be discovered by cDNA library screening [59,60]. Since then, many neoantigens from single-nucleotide variants have been identified by the cDNA library screening approach, which were summarized previously [61]. Thanks to the development of next-generation sequencing, the identification of neoantigens from single-nucleotide variants has become quite efficient. The development of these approaches will be discussed in Section 3.

### 2.2. Insertion, Deletion and Fusion

In addition to nonsynonymous SNV, other alternations in the genomic DNA, including insertion, deletion and fusion, also lead to the changes in protein sequences, which and are also potentially recognized by T cells [62]. Some of the insertions and deletions lead to changes in reading frames, which are called frameshift mutations. Frameshift mutations generate long peptides that differ from normal peptides. In a personalized neoantigen vaccine study, six patients with melanoma received pools of synthetic long peptides derived from neoantigen sequences, including frameshift mutations. Eleven neoantigens generated from frameshift mutations could be recognized by the T cells in patients’ peripheral blood after vaccination [63].

Chromosome abnormalities in tumor cells, such as chromosome translocation, may result in a fusion gene which contains exons from part of gene A and part of gene B. A mutated peptide may possibly be generated at the junction site. Neoantigens generated from fusion genes have not been reported in melanoma. However, an interesting report showed that the product of an in-frame DEK–AFF2 fusion could be recognized by T cell populations isolated from the peripheral blood of a patient with head and neck squamous cell carcinoma [64]. DEK–AFF2 fusion-reactive T cells were likely responsible for the complete response of the checkpoint inhibitor therapy observed in this patient. Therefore, fusion genes may potentially generate neoantigens.

### 2.3. Post-Transcriptional and Post-Translational Alternations

Some of the alternations at the post-transcriptional step may generate different protein sequences in tumor cells, but not in normal cells. Very frequently, new proteins which are not expressed in normal cells may be produced by tumor cells through the mechanism of alternative splicing. In a comprehensive study, 8705 tumor specimens from 32 cancer types were analyzed, and about 930 alternative splicing events on average were detected in tumors, but not in normal samples [65]. Alternative splicing may potentially serve as an important source for neoantigens [66]. Additionally, alternative start codons or reading frames may potentially generate neoantigens as well. A classic example is the NA-17A antigen. NA-17A peptide was generated from an intron region in the N-acetylglucosaminyltransferase V gene, which was likely the result of an alternative start site or an alternative splicing event. NA-17A can be detected in multiple melanoma cell lines, and can be recognized by a T cell clone isolated from melanoma tumor-infiltrating lymphocytes (TILs) [67,68].

Another major source of post-transcriptional modification is adenosine (A) to inosine (I) RNA editing. ADAR (adenosine deaminase acting on RNA) enzymes may edit A to I at specific nucleotide sites. Inosine may pair with cytidine, just as guanosine pairs with cytidine, resulting in altered proteins at the translational step [69,70]. These altered proteins may potentially be processed and presented by MHC molecules on the cell surface. Zhang M et al. reported that peptides from edited cyclin I could be presented on the surface of melanoma cells and detected by TILs [71].

## 3. Approaches to the Identification of Neoantigens

### 3.1. cDNA Library Screening

The classical approach to the identification of neoantigens utilized cDNA library screening. A cDNA library was made from a melanoma cell line. Pools of cDNA were transfected into a cell line, and then the cells would present the potential neoantigens to T cells. Neoantigens that were recognized by the T-cells were considered positive hits, and these were further studied to isolate the cDNA clones encoding the neoantigens. Nonsynonymous SNVs in the CDK4, MUM1 and CTNNB1 genes were among the first neoantigens isolated from melanoma by this approach [59,60,72]. Other neoantigens identified by this approach were summarized previously [61]. However, it could take months to identify a neoantigen using the cDNA library screening approach. With the recent advances in technology, this approach has not been actively used in recent years.

### 3.2. Neoepitope Prediction

The approach of using predicted neoepitope to identify neoantigen was initiated by Matsushita et al. [73]. Nonsynonymous mutations in a mouse sarcoma cell line were identified by whole-exome sequencing. Amino acid sequences near the mutation sites were subjected to an in-silico analysis to identify potential epitopes that could strongly bind to the MHC class I molecule, H-2D or H-2K. Mutated Sptbn1 was then identified as a potential tumor-rejection antigen. In a similar approach, nonsynonymous mutations were identified from melanoma specimens resected from three patients who received adoptive cell therapies with TILs [74]. TILs were stimulated with pools of predicted neoepitopes based on MHC class I binding affinities, and seven neoantigens from these three melanoma specimens were identified by this approach. These neoantigens were validated by stimulating TILs with neoepitopes presented by HEK293 or the COS-7 cell line, and the activation of neoantigen-reactive T cells was detected by IFN-γ ELISA or an ELISpot assay. Another method to validate neoepitopes was MHC-tetramer staining. TILs were stained with potential neoepitope-tetramer complexes. Positive staining by flow cytometric analysis was considered as a neoantigen-reactive T cell population [75,76].

This approach relies on the accuracy of prediction algorithms, which have been improved significantly in recent years. The latest effort was conducted by the Tumor Neoantigen Selection Alliance [77]. Twenty-eight teams utilized their own bioinformatics pipelines to predict neoepitopes from six specimens, including three from metastatic melanoma and three from non-small cell lung cancer. By combining the strengths in several pipelines, a PRNA (predicted and recognized neoantigen abundance) metric was developed that prioritized several features, including strong MHC binding affinity, high tumor abundance, high MHC binding stability, and peptide recognition. This PRNA metric could filter out 98% of non-immunogenic peptides, with a precision of over 0.70.

The prediction approach has the advantage of analyzing dozens of samples within a few weeks, and it has been widely used to study the association between the number of neoantigens and the outcomes of checkpoint blockade therapy, as discussed in Section 5.2. On the other hand, the prediction is not completely accurate, and there is still room for improvement. The uncertainty of prediction has to be clearly stated, otherwise the usage of “predicted” neoantigens, rather than “validated” neoantigens, might jeopardize the integrity of scientific findings.

### 3.3. Tandem Minigene and Long-Peptide Libraries

In order to overcome the imperfection of neoepitope prediction algorithms, a tandem minigene approach was developed [78]. Nonsynonymous mutations, mostly SNVs, were identified by whole-exome sequencing for tumor specimens. Each minigene contained an identified mutated amino acid, flanked by 12 normal amino acids on both the N- and C-termini. This minigene could cover all of the different possibilities of neoepitopes ranging from eight to 13 amino acids in length. Next, six to 24 minigenes were joined in tandem, and the cDNA encoding a tandem minigene was synthesized and cloned into an expression vector. A tandem minigene library was then transfected into a cell line to present all of the potential neoepitopes/neoantigens to T cells. Mutated KIF2C and POLA2 neoantigens were identified from two melanoma specimens in the initial study. Similarly to the tandem minigene approach, a synthetic long peptide, corresponding to a minigene, could be used to assemble a neoantigen library for screening. These approaches have been used in several studies [79,80,81]. More significantly, this design, with some minor modifications, has been utilized to develop personalized neoantigen vaccines for patients with melanoma [63,82,83].

### 3.4. HLA Peptidomics

One of the concerns about neoantigen identification is that the predicted neoepitopes may not naturally present on the MHC/HLA molecules. In order to study the peptides presented on HLA molecules, a HLA peptidomics technique may be utilized. In this technique, tumor cells are lysed and HLA-peptide complexes are purified by immunoprecipitation. Lastly, peptides are eluted from the HLA molecules and analyzed by mass spectrometry. Additionally, mutations identified by whole-exome sequencing may be used to assist in the identification of neoepitopes [84,85]. Using this approach, five neoantigens were identified in three patients with melanoma in a study [84]. Additionally, two of the neoantigen-reactive T cell clones found in this study could kill most of the autologous melanoma cells both in vitro and in vivo. This approach may have a strong potential to accurately identify neoantigens in the future.

## 4. Clinical Trials for Melanoma Immunotherapy

In most cases, the development of therapeutic agents has been uni-directional, from basic research, to translational research, and then finally to clinical trials. However, the development of melanoma immunotherapy was bi-directional, and the success in clinical trials has paved the way for more hypothesis-driven research, especially in the areas of neoantigens and T cells. Here, we describe the sentinel immunotherapy clinical trials for cutaneous melanoma, and demonstrate how these trials have drastically improved the standard of care for patients with stage III/IV melanoma (Table 1 and Table 2). In the next section, we will discuss the basic and translational research in neoantigens and T cells, which were built based on the solid observations in clinical trials.

### 4.1. Monotherapy

Ideally, patients diagnosed with cutaneous melanoma may be treated and cured with surgery alone. Many patients with early-stage melanoma, and even some with later stages of melanoma, are indeed cured with surgery. However, recurrence occurs among patients with all stages of cutaneous melanoma, even despite the best oncologic surgery. The development of immunotherapy has remarkably changed the way in which patients with metastatic melanoma or a high risk of recurrence are treated, and has led to both durable responses and cures in patients who did not have effective treatment options less than a decade ago.

#### 4.1.1. Cytokines

Prior to 2011, the only available therapies for melanoma included high-dose interferon (IFN) in the adjuvant setting for patients with a high risk of recurrence, and dacarbazine for patients with unresectable or metastatic disease. The prognosis for such patients was poor, as the response rates were low, along with high systemic toxicity for patients treated with IFN or dacarbazine. In 1996, the groundbreaking E1684 trial was published, which randomized patients with high-risk stage II and III melanoma to high-dose interferon α-2b (HDI) versus observation after the surgical resection of their disease. The patients were treated for 1 year after surgery, and those in the HDI group showed statistically significant relapse-free survival (RFS, from 1 to 1.7 years) and overall survival (from 2.8 to 3.8 years) [86]. Unfortunately, constitutional, hematologic, and neurologic toxicities were common with HDI, and dose delays or dose reductions occurred in 72% of the patients, therefore limiting its use for melanoma [86]. Simultaneously, high-dose IL-2 was developed for patients with unresectable regional or metastatic melanoma. As early as 1985, patients with metastatic melanoma were treated with intravenous IL-2 [87]. Treatment-related toxicities are common with systemic IL-2 therapy, with the most severe resembling the clinical manifestations of septic shock, including hypotension, tachycardia, and even acute respiratory distress syndrome [88]. The patients required minimal to no medical comorbidities and excellent performance status in order to receive such treatment [88], and this was available at very few centers in the United States. Despite high toxicity, the effects with systemic IL-2 are striking, as drastic responses occurred even in patients with a large metastatic tumor burden. What was also unique about these responses was the durability. The median progression-free survival (PFS) was 13.1 months in patients with only partial responses. In patients with a complete response, the median duration of response was not reached at 40 months, when the study was reported. Ten of the 17 complete responders remained disease-free [88]. These durable responders established that it is possible to re-active patients’ immune systems against cancer. Despite the durability offered by systemic IL-2, the therapies required administration in a specialized center. Soon after, alternative immunotherapies were developed that would become widely available, both geographically and to patients with comorbidities. Table 1 lists the current FDA-approved immunotherapies for patients diagnosed with stage III/IV cutaneous melanoma. Targeted therapies with BRAF and MEK inhibitors are also listed on this table for reference.

#### 4.1.2. Checkpoint Blockade Antibodies

Ipilimumab is a monoclonal antibody designed to bind to the co-inhibitory molecule CTLA-4 and block the interaction between CTLA-4 and its ligand CD80/CD86. Anti-tumor T cells are re-activated following the administration of the anti-CTLA-4 checkpoint blockade antibody [38]. Ipilimumab was the first of its kind to be approved as an agent against unresectable stage III or stage IV metastatic melanoma. Clinical trial CA184-002 included patients with unresectable stage III or IV melanoma whose disease had progressed on standard-of-care systemic therapy. The patients were randomly assigned in a 3:1:1 ratio to receive ipilimumab plus gp100, ipilimumab alone, or gp100 alone. An improved response rate, response duration, PFS, and overall survival (OS) occurred in patients who received ipilimumab, regardless of the addition of a cancer vaccine targeting melanoma antigen gp100 [89]. A second study, CA184-024, looked at high-dose ipilimumab at 10 mg per kg plus dacarbazine to dacarbazine and a placebo in patients with previously untreated metastatic melanoma. This study found higher OS rates in the ipilimumab plus dacarbazine group at 1 year (47.3 vs. 36.3% *p* < 0.001) and 3 years (20.8 vs. 12.2%, *p* < 0.001) [90]. An additional study, CA 184-025, compared ipilimumab to systemic dacarbazine, which was at that time the gold-standard treatment. Extended follow up showed that ipilimumab resulted in long-term survival in approximately one-fifth of patients, with a 5-year OS of 18% vs. 9% for dacarbazine [91], again proving that immune system manipulation may lead to impressive anticancer activity. Adverse events do occur with ipilimumab, and these effects are dose-dependent [92].

Pembrolizumab is another checkpoint blockade antibody that binds to PD-1 on T cells and blocks the interaction between PD-1 and its ligand PD-L1/L2. The phase II randomized trial KEYNOTE-002 evaluated pembrolizumab in patients with metastatic melanoma which was refractory to ipilimumab. Pembrolizumab improved the response rates and PFS compared with chemotherapy [93]. The most common grade 3–4 adverse events were fatigue, hypopituitarism, colitis, diarrhea, hyponatremia, and pneumonitis [93]. The phase III trial KEYNOTE-006 directly compared pembrolizumab 10 mg/kg to ipilimumab 3 mg/kg in patients with metastatic melanoma who were naïve to either treatment. The patients enrolled had BRAF V600 mutation-positive unresectable stage III or IV melanoma, and had received up to one previous round of systemic therapy for advanced disease; therefore, this population was not heavily pretreated. The patients in the pembrolizumab group had a longer median PFS of 8.4 months versus 3.4 months in the ipilimumab group. This study did show a significant survival benefit of pembrolizumab over ipilimumab, as the median overall survival was 32.7 months in the combined pembrolizumab groups compared to only 15.9 months in the ipilimumab group, after a median follow up of 57.7 months. The patients who received pembrolizumab also experienced fewer treatment-related adverse events [94]. Nivolumab is also an anti-PD-1 monoclonal antibody which was developed and studied simultaneously with pembrolizumab, with similar results showing its efficacy over systemic chemotherapy and ipilimumab in the phase III CheckMate-066, where patients receiving nivolumab had a higher 3-year OS compared to chemotherapy, and a longer median OS of 37.5 months compared to 11.2 months in the dacarbazine group [95]. The CheckMate-067 study also demonstrated that nivolumab has higher response rates than ipilimumab [94]. Nivolumab has a similar side effect profile to pembrolizumab [95]. Taken together, the landmark trials for pembrolizumab and nivolumab led to a change in the standard of care. With FDA approval, anti-PD-1 antibodies became the first-line treatment for advanced melanoma over ipilimumab, dacarbazine, and high-dose IL-2 due to their superior response rates, durability, and lower rates of systemic toxicity.

In addition to PD-1, an alternative approach is to target its natural ligand, PD-L1, which is expressed on some tumor cells and some antigen-presenting cells. In the clinical trial for atezolizumab, the median OS was 23 months among 43 melanoma patients, including 33 patients with cutaneous melanoma [96]. In a trial for avelumab, the median OS was 17.2 months in a subset of 35 melanoma patients, including 28 patients with cutaneous melanoma [97]. In general, the response rates in anti-PD-L1 antibody clinical trials were less favorable compared to the anti-PD-1 antibodies, and these antibodies have not received approval by the FDA as monotherapy agents. More encouraging clinical outcomes have been observed in combination therapies with other drugs, such as an atezolizumab + vemurafenib + cobimetinib combination therapy (Section 4.2; Table 2), which will be the focus for future development.

#### 4.1.3. Other Therapeutic Agents

Talimogene laherparepvec (T-VEC) is an attenuated oncolytic herpes simplex virus that contains the granulocyte macrophage colony-stimulating factor (GM-CSF) gene. The intralesional injection of this oncolytic virus is uniquely designed to specifically target and destroy cancer cells whilst sparing normal tissue. Its other benefits include limited systemic toxicity and the theoretical potential to induce a systemic antitumor immune reaction, along with the regression of noninjected tumors [98]. The OPTiM trial enrolled patients with injectable unresectable in-transit disease, comprising patients with stage IIIB/C/IV melanoma. This trial demonstrated an increase in the durable response rate of 23.3% in individuals who received T-VEC compared to the 2.1% rate for placebo GM-CSF [98]. In total, 10% of patients experienced complete responses to T-VEC. The median OS was not statistically different between the groups. The side effects were minimal, including fatigue, chills, and pyrexia [98]. The final analysis also confirmed that T-VEC was associated with lasting complete responses, as 88.5% of those with complete responses survived at 5 years [99,100]. Trials are ongoing regarding the evaluation of T-VEC in combination with systemic PD-1 inhibitors for advanced melanoma which is refractory to the systemic PD-1 inhibitor alone, with the hypothesis that combining immunotherapeutic agents may enhance the efficacy, and may also lead to responses in patients who fail to respond to PD-1 monotherapy [101].

As reviewed, IL-2 is a systemic therapy, but it may also be used as an intralesional therapy for unresectable in-transit melanoma which is refractory to systemic immunotherapy [102,103]. A 2014 meta-analysis on intralesional IL-2 found that 67–96% of the injected lesions exhibited a complete response. Many of these were durable [102]. Notably, intralesional IL-2 is well tolerated. Common side effects include a local inflammatory reaction at the site of injection, fatigue, and chills, which resolve with analgesics [102]. There are other intralesional therapies which are being studied in patients with refractory in-transit disease, including Bacillus Calmette-Guerin (BCG) and Rose Bengal [104,105].

Although they are not a form of immunotherapy, the development of BRAF inhibitors of the MAP kinase pathway was also groundbreaking for the treatment of advanced melanoma. These inhibitors are now used in combination with immunotherapy, and therefore will be reviewed briefly. Vemurafenib and dabrafenib are both BRAF inhibitors that may be used to treat individuals with a common mutation V600E in the BRAF gene. Approximately 40% of patients with metastatic melanoma harbor this mutation, and thus are eligible for treatment with this class of therapy [57,58]. These drugs were studied for metastatic melanoma in large trials between 2010 and 2012, just prior to the FDA approval of ipilimumab and then PD-1 inhibitors for melanoma. The phase III trials BRIM-3 and BREAK-3 compared BRAF inhibitors to dacarbazine in patients with previously untreated stage IV or unresectable stage III BRAF V600E-positive melanoma, showing a BRAF inhibitor improves response rates, PFS, and OS compared with chemotherapy alone [103,104]. The long-term analysis of BREAK-3 demonstrated durability with this treatment, with 11–12% remaining progression free at 5 years [106]. This durability does not match that of immunotherapy, as the majority of responses to BRAF inhibitors are short-lived. A subgroup analysis of pooled data from the pembrolizumab KEYNOTE-001, -002, and -006 trials evaluated patients with advanced melanoma and known BRAF V600E/K tumor mutations who had received pembrolizumab. The patients with BRAF mutations had similar responses to immunotherapy to patients with wild-type mutations [107]. The outcomes from this analysis support the use of pembrolizumab as a first-line treatment of advanced melanoma regardless of its BRAF V600E/K mutation status [107].

### 4.2. Combination Therapy

Although the results from monotherapy treatment for stage III unresectable or stage IV melanoma were promising, there were many patients who did not respond, and resistance to therapy developed in many of the responders. This led to the development of combination therapy for melanoma. Early combination therapies included BRAF and MEK inhibitors, targeting two points on the MAP kinase pathway, and then BRAF/MEK inhibition with immunotherapy, in order to prevent or delay the onset of resistance observed with MAP kinase inhibitors alone [108,109]. The key findings of these trials are summarized on Table 2, and demonstrate longer PFS and OS compared to monotherapy. Toxicities when combining BRAF/MEK inhibitors and immunotherapy make these therapies difficult for patients to complete [110,111].

As was previously mentioned, the CheckMate-067 and CheckMate-069 trials analyzed the combination of ipilimumab and nivolumab in previously untreated unresectable stage III or stage IV disease. When compared to monotherapy, the combination group had an improved response rate and PFS. The results from CheckMate-067 demonstrated a five-year OS of 52%, 44%, and 26% in the nivolumab/ipilimumab, nivolumab, and ipilimumab groups, respectively [112]. CheckMate-069 also demonstrated that PFS was longer with combination therapy than with nivolumab alone, resulting in 11.2 months versus 5.3 months [110,111]. In the trial IMspire150, a triple regimen—atezolizumab, vemurafenib and cobimetinib—was used to treat patients with BRAF mutations in a randomized trial. The PFS was 15.1 months versus 10.6 months in the atezolizumab placebo arm [113]. It became the only triple regimen approved by the FDA thus far.

### 4.3. Adjuvant Therapy

Patients with Stage III melanoma have a high risk of recurrence, even after the complete resection of all known disease. Treatment with modern adjuvant therapy has been shown to decrease the risk of recurrence in patients with stage IIIB, IIIC, and IIID disease, and will be reviewed in this section. Of note, these clinical trials were performed under the AJCC 7th edition staging for melanoma, whereas the AJCC 8th edition is now in use, in which changes were made where some patients with stage IIIA disease in the 7th edition are now considered to have stage IIIB disease under the 8th edition, and vice versa. The criteria in the adjuvant trials typically included a sentinel lymph node disease burden of at least 1 mm in size. There are no FDA-approved modern adjuvant therapies for patients with stage I or II melanoma, including the high-risk stage IIC group. Clinical trials are currently underway for stage IIC patients, as these patients actually have a higher risk of melanoma recurrence compared to patients with stage IIIA melanoma [114]. Lastly, patients with stage IIIA (AJCC 8th edition) melanoma have a long overall survival without adjuvant therapy, and the use of adjuvant therapy in this group is for selected stage IIIA patients with the highest risk of recurrence, because the clinical benefit of adjuvant therapy is low in this group of patients.

Ipilimumab was studied in the adjuvant setting on the EORCT 18071 trial, where patients with stage III melanoma were randomized to high-dose ipilimumab at 10 mg/kg versus a placebo for up to 3 years. The 3-year recurrence-free survival was 46.5% in those receiving ipilimumab versus 34.8% in the placebo. Adverse events occurred frequently at this high dose, with 52% discontinuing the treatment due to side effects, and five patients died of drug-related toxicity [115]. The CheckMate-238 trial compared adjuvant nivolumab to adjuvant ipilimumab at 10 mg/kg for one year of treatment. Nivolumab improved RFS compared to ipilimumab, and there were significantly fewer side-effects in the nivolumab group; again, treatment-related deaths occurred in the ipilimumab group [116]. The KEYNOTE-54 trial compared adjuvant pembrolizumab versus a placebo, and pembrolizumab improved RFS. There was one treatment-related death in the pembrolizumab group [117].

Based on these trials, high-dose ipilimumab, pembrolizumab, and nivolumab are all FDA-approved for the adjuvant treatment of patients with stage III melanoma with a high risk of recurrence. These drugs were approved by the FDA based on recurrence-free survival, as they have not yet shown a disease-specific survival benefit with adjuvant therapy. Of note, BRAF inhibitors have also been studied and approved by the FDA in the adjuvant setting based on improved recurrence-free survival [118,119], but typically this class is selected only if PD-1 inhibitors are contraindicated. With the approval of these drugs, interferon is no longer used in the adjuvant setting.

### 4.4. Neoadjuvant Therapy

Because there are high rates of tumor responses with immunotherapy, clinicians hypothesize that it may be better used in the neoadjuvant setting. There are several potential reasons for the neoadjuvant administration of immunotherapy, e.g., prior to therapeutic lymph node dissection for bulky nodal disease, it may be beneficial over adjuvant therapy. These include the optimization of oncologic resection with minimal morbidity, allowing time to test the tumor biology in those patients who may already have undetectable microscopic distant disease, and predicting the therapeutic response and toxicities, thus optimizing the selection of additional therapy. In addition, neoadjuvant therapy may allow for a more robust immune response compared to adjuvant therapy due to an intact tumor microenvironment [120,121]. Simultaneously, there is the risk of losing the window of opportunity for surgery in non-responders selected for neoadjuvant therapy. There are many ongoing clinical trials evaluating these theories and patient outcomes, as listed in Table 3.

In a phase II trial, neoadjuvant anti-PD-1 treatment with up to four cycles of nivolumab in patients with resectable stage III/IV melanoma led to tumor responses in 25%. In this same trial, the neoadjuvant combination of ipilimumab and nivolumab lead to tumor responses in 73%. However, the toxicity was high, with 73% of the patients also experiencing a grade 3 adverse event. Only 8% of the patients in the nivolumab-only arm experienced grade 3 adverse events [122]. There were a remarkably high number of pathologic complete responses. The OpACIN trial also looked at the use of neoadjuvant ipilimumab plus nivolumab in individuals with macroscopic stage III melanoma. The study found that two cycles of neoadjuvant ipilimumab plus nivolumab without additional adjuvant therapy induced durable regression-free survival in more than 80% of patients [123,124].

**Table 3:** Current clinical trials on neoadjuvant therapy for stage iii melanoma.

Drug	Trial/ID	Dosage	Primary Outcome/ Estimated Completion Date
Pembrolizumab	NCT02434354	200 mg Pembro followed by surgery, then adjuvant Pembro therapy every 3 weeks for 1 year	July 2022
Ipilimumab + nivolumab	OpACIN NCT02977052	Arm A: 3 mg/kg Ipi + 1 mg/kg Nivo every 3 weeks for 2 cycles prior to surgery. Arm B: 1 mg/kg Ipi + 3 mg/kg Nivo every 3 weeks for 2 cycles prior to surgery Arm C: 3 mg/kg Ipi every 3 weeks for 2 cycles, followed immediately by 3 mg/kg Nivo every 2 weeks for 2 cycles prior to surgery	June 2025
Dabrafenib+ trametinib	NCT01972347	150 mg Dab + 2 mg Tram for 12 weeks followed by surgery, then 40 weeks of adjuvant Dab/Tram	May 2022
Ipilimumab	NCT00972933	Two doses of 10 mg/kg of Ipi followed by surgery, then two doses of adjuvant Ipi	Median PFS: 11 months [125]
Nivolumab vs. Ipilimumab + nivolumab vs. Nivolumab + relatlimab	NCT02519322	Arm A: 3 mg/kg Nivo every 2 weeks for 4 cycles prior to surgery, then adjuvant Nivo every 2 weeks for 13 cycles Arm B: 1 mg/kg Nivo and 3 mg/kg Ipi every 3 weeks for 3 cycles prior to surgery, then adjuvant Nivo every 2 weeks for 13 cycles Arm C: 480 mg Nivo + 160 mg relatlimab every 4 weeks for 2 cycles prior to surgery, then adjuvant Nivo + relatlimab every 4 weeks for 10 cycles	December 2022

Abbreviations: Dab—dabrafenib; Tram—trametinib; Nivo—nivolumab; Ipi—ipilimumab; Pembro—pembrolizumab.

## 5. The Role of Neoantigens in Immunotherapy

### 5.1. Animal Models

The role of neoantigens in immunotherapy has been studied well in mouse models. The first study was conducted to identify neoantigen Sptbn1, as described in Section 3.2. [73]. The mutated Sptbn1 was detected in a methylcholanthrene (MCA)-induced sarcoma cell line d42m1-parental, which was partially rejected after it was transplanted into naïve wild-type mice. However, some subpopulations of d42m1 could escape from immune surveillance, and mutated Sptbn1 was not detected in these populations. In addition, mutated Sptbn1-specific CD8^+^ T cells were detected in tumors and draining lymph nodes in tumor-bearing mice. These results suggested that the neoantigen Sptbn1 played a role in immune responses against tumors. In another study, 962 nonsynonymous mutations were identified in a commonly used murine melanoma cell line, B16F10 [126]. The top 50 mutations were further characterized, based on expression and epitope prediction. In total, 50 long peptides encoding these mutations were injected into mice, and one third of them induced responses against the mutations. Lastly, a peptide vaccine targeting mutated Kif18b could control the growth of B16F10 in vivo.

In a mouse model of checkpoint blockade immunotherapy, d42m1 clone T3 tumor cells could grow in wild-type mice, but were rejected following anti-PD-1 or anti-CTLA-4 immunotherapy [127]. Mutated Alg8 and mutated Lama4 were identified as neoantigens recognized by CD8^+^ T cells after anti-PD-1 immunotherapy. D42m1-T3 tumors could also be rejected in mice following a vaccine treatment containing long peptides encoding mutated Alg8 and mutated Lama4. In a subsequent study, mutated Itgb1-specific CD4^+^ T cells were found to be required for optimal immune responses following checkpoint blockade immunotherapy or vaccine therapy [128]. Therefore, in addition to CD8^+^ T cells, neoantigen-reactive CD4^+^ T cells also played an important role in immune responses against tumors.

### 5.2. Checkpoint Blockade Immunotherapy

Although the role of neoantigen-reactive T cells in immunotherapy has been shown in animal models, this hypothesis is more difficult to test in humans. Several studies utilized prediction algorithms to predict the neoepitopes generated from each tumor, in the attempt to discover correlations between the predicted neoepitopes and clinical responses. In the first study, whole-exome sequencing was performed to identify mutations from tumors, which were obtained from patients with melanoma who underwent CTLA-4 checkpoint blockade immunotherapy. The predicted neoepitopes and numbers of mutations (also known as the tumor mutational burden, TMB) were strongly associated with long-term clinical benefits in both the discovery set (*n* = 25) and the validation set (*n* = 39) [129]. In another similar study, TMB and the predicted neoepitopes were also significantly associated with clinical benefits for 110 patients who were treated with CTLA-4 checkpoint blockade immunotherapy [130].

In the studies for patients after PD-1 checkpoint blockade immunotherapy, TMB was significantly associated with survival in the ipilimumab-naïve patient group (*n* = 33), but not the ipilimumab-treated patient group (*n* = 35) [131]. In another study with 38 patients, TMB, but not predicted neoepitopes, were significantly correlated with improved patient survival [132]. Although the predicted neoepitopes were higher in the responder group, the difference was not statistically significant. Additional evidence was provided in support of the correlations between TMB and clinical benefits, but predicted neoepitopes were not studied in these publications [133,134]. Lastly, the Tumor Neoantigen Selection Alliance was assembled with 28 international teams, as mentioned in Section 3.2 [77]. One of the major goals of the alliance was to optimize the prediction algorithm, such that it could potentially predict the outcomes of immunotherapy. A cohort of 55 patients with melanoma was treated with PD-1 checkpoint blockade immunotherapy. Using the PRNA algorithm for neoepitope prediction, the overall survival of the patients with above-median PRNA was longer than those with below-median PRNA (*p* = 0.063). Besides melanoma, a pioneer study came from an immunotherapy clinical trial for patients with mismatch repair-deficient cancers [135]. The hypothesis was that mismatch repair-deficiency could generate a large number of somatic mutations, leading to more neoantigens. Nine out of 17 patients with mismatch repair-deficient cancers responded to PD-1 checkpoint blockade immunotherapy. On the other hand, none of the 18 patients with mismatch repair-proficient colorectal cancer responded to the therapy. In a follow-up study, an objective response rate of 29% was observed in 102 patients with TMB-high tumors. This led to the FDA approval of pembrolizumab for patients with TMB-high tumors in 2020 [136]. Taken together, although some correlations were established between predicted neoepitopes and clinical benefits, the results were not consistent across different studies. The small cohorts with large diversities in tumor types, as well as the imperfect neoantigen prediction algorithms, may have contributeed to these inconsistent results.

### 5.3. Adoptive Cell Therapy (ACT)

ACT is another type of immunotherapy that directly transfers cells into patients. One approach is to genetically modify peripheral blood T cells to re-direct the specificities of T cells. For example, peripheral blood T cells were genetically modified with a TCR that recognized a melanocyte differentiation antigen, MART-1. Two out of 17 patients experienced partial responses in the initial clinical trial [137]. However, because MART-1 was also expressed on normal melanocytes in the skin, eye, and ear, the targeting of MART-1 also induces unwanted toxicities, including vitiligo, uveitis and hearing loss [46]. In another TCR clinical trial targeting the cancer-germline antigens MAGE-A3, A9 and A12, nine patients, including seven patients with metastatic melanoma, were treated with TCR-engineered T cells at a high dose [138]. Objective clinical responses were observed in five patients with melanoma. However, two patients experienced severe neurological toxicities and died. The toxicities were likely due to the expression of MAGE-A12 in the brain. Therefore, a more tumor-specific TCR may be less likely to induce unwanted toxicities such as those described. In a phase I/II clinical trial, purified CD4^+^ T cells were genetically modified with a highly specific TCR recognizing MHC class II-restricted MAGE-A3 and A6 epitopes. These modified T cells were used to treat 17 patients with metastatic cancers, including six patients with cutaneous melanoma and one patient with mucosal melanoma [29]. Objective responses were observed in one patient in a low-dose cohort and three patients in the highest-dose cohort, but no patients with melanoma responded to this treatment. One patient with breast cancer experienced severe toxicities, with renal and liver toxicities. In the future, it is critical to identify more TCRs that may specifically recognize melanoma and other solid-tumor cells [139].

In another approach, TILs were grown in vitro from tumors resected from patients with metastatic melanoma. These TILs were greatly expanded and transferred back to the patients [140]. The response rates ranged up to 72% in several clinical trials, and approximately 20% of these patients experienced long-term, complete tumor regressions [141,142,143,144,145]. These patients were disease free after long-term follow-up. The current hypothesis is that neoantigen-reactive T cells within the TIL population play a major role in T cell-mediated tumor regressions [55,56,146]. This is based on an initial observation in a patient with metastatic melanoma who was treated with ACT-TIL and experienced a complete response for more than a decade [147]. More than 50% of the TILs reacted to a neoantigen PPP1R3B, and no reactivity against non-mutated antigens was detected. The neoantigen-reactive T cells persisted in the patient’s peripheral blood for more than 5 years following the ACT-TIL therapy. Additionally, strong neoantigen reactivities were detected in TIL products, which have been used for successful ACT-TIL therapies for patients with metastatic melanoma. These studies included three TIL products detected by neoepitope prediction and two TIL products detected by tandem minigene library screening [74,78]. Besides melanoma studies, evidence on neoantigens was also obtained from ACT-TIL therapies for patients with gastrointestinal or breast cancers [81,148,149]. Because the TILs were mixed populations, there is the possibility that other tumor-reactive T cells, such as MART-1-specific T cells, might also play a role in tumor regressions [150]. The definitive answer will come from ATC using genetically modified T cells targeting neoantigens, especially targeting shared neoantigens derived from hotspot mutations [56,61,151,152].

### 5.4. Neoantigen Vaccine

Another potentially impactful development is personalized neoantigen vaccines. The first study for these utilized a dendritic cell vaccine. Peptides encoding predicted neoepitopes were pulsed on autologous dendritic cells, which were then injected intravenously into three patients with stage III melanoma. Neoantigen-reactive T cells in the peripheral blood were strongly induced after vaccination in all three patients, but no clinical response was observed in this study [79]. In another study, an RNA vaccine encoding the predicted neoepitopes was used to treat patients with metastatic melanoma [82]. In addition to strong T-cell responses against neoantigens, two of the five patients experienced vaccine-related objective responses. One additional patient experienced a complete response after the vaccine and pembrolizumab treatments. In the third study, six patients with metastatic melanoma were treated with a peptide vaccine targeting the predicted neoepitopes [63]. Four of these patients had no recurrence after vaccination. Two patients with recurrent diseases were subsequently treated with pembrolizumab and experienced complete responses. A follow-up study from this group indicated that the neoantigen vaccine could induce the long-term persistence of neoantigen-reactive T cells [83]. They also observed tumor infiltration by neoantigen-reactive T cells and epitope spreading. Taken together, a personalized neoantigen vaccine may potentially become an effective treatment in combination with checkpoint blockade immunotherapy.

### 5.5. The Dysfunction and Re-Activation of Neoantigen-Specific T Cells

It is well-known that neoantigen-specific T cells are continuously stimulated by neoantigens in the tumor microenvironment, leading to T cell dysfunction, exhaustion and death [153]. A recent single-cell study highlighted this observation. Thirty neoantigen-specific TCRs were isolated from the tumors and blood of four melanoma patients, who underwent the neoantigen vaccine trial (Section 5.4). Single-cell transcriptome analysis revealed that T cells with neoantigen-specific TCRs showed a transcription profile associated with exhaustion, including exhaustion markers PD-1 and CD39, as well as low levels of memory markers IL7R and TCF1 (also known as TCF7) [154]. On the other hand, we cannot ignore the fact that checkpoint blockade antibodies are still capable of re-activating T cells and inducing tumor regressions, as shown by the clinical responses. In the attempts to address this, scientists proposed two different hypotheses. In the first hypothesis, dysfunctional, exhausted T cells may contain two subsets, the “early” exhaustion, Eomes^lo^PD-1^int^Tcf1^+^ subset and the “terminal” exhaustion Eomes^hi^PD-1^hi^Tbet^lo^Tcf1^−^ subset [155]. The early exhaustion subset still has some proliferative capacity, but has limited cytotoxicity. On the other hand, the terminal exhaustion subset has a poor proliferative capacity but still has some cytotoxicity [156]. After antigen stimulation, T cells in the early exhaustion subset may proliferate and maintain the current status, or they may gradually convert to the terminal exhaustion subset. The transition from early to terminal exhaustion status is irreversible. Importantly, PD-1 checkpoint blockade may reinvigorate the early exhaustion subset, but not the terminal exhaustion subset [157].

The second hypothesis proposes that some rare populations of neoantigen-specific T cells maintain stem- or memory-like properties, and they may proliferate and activate following viral infection or checkpoint blockade immunotherapy. The major evident to support this hypothesis is the identification of a T-cell population expressing TCF1 [21,22,23,24,25]. In addition to mouse models, TCF1^+^PD-1^+^ T cells were also detected in tumors and peripheral blood isolated from patients with melanoma [24]. Upon antigen stimulation, these stem-like TCF1^+^ T cells underwent self-renewal, and also differentiated into terminally differentiated, exhausted T cells. Similarly, a recent study suggested that a CD39^−^CD69^−^ population within neoantigen-specific TILs showed stem-like properties, and higher percentages of CD39^−^CD69^−^ populations in TILs were associated with better clinical responses following the ACT-TIL therapies [158]. Lastly, neoantigen-specific TILs were isolated from patients with non-small cell lung cancer who underwent neoadjuvant PD-1 therapy, and single-cell TCR and transcriptome analyses were performed [159]. In favor of the second hypothesis, neoantigen-specific TILs from major pathologic responders expressed higher levels of genes associated with effector function and memory, such as TCF1, compared to non-responders. The authors further hypothesized that neoantigen-specific TILs from non-responders expressed low avidity or affinity TCRs, leading to unfavorable transcriptional programs. However, the expression levels of tumor antigens and tumor microenvironments may also strongly influence the T cell differentiation, leading to different observations [154,160]. As a result, the further investigation of this hypothesis is required.

### 5.6. Neoantigen-Specific T Cells: A Minor Population in TILs

In addition to the quality of neoantigen-specific T cells, the quantity of these T cells is also in question. A pioneer study suggested that the majority of tumor-infiltrating T cells were bystanders, including viral-specific T cells, and neoantigen-specific T cells only represented a small population [75]. However, with the small numbers of patients, as well as the difference between methodologies, it is difficult to estimate the total numbers of neoantigen-specific T cells in TILs in general. In an initial study for melanoma, the top 10 most-frequent TCRs from tumor-infiltrating CD8^+^PD-1^+^ populations were isolated and tested for their specificities. Using this approach, the frequencies of neoantigen-specific TCRs in TILs ranged from 0.18% to 7.25% in five metastatic melanoma specimens [80]. In our recent study, PD-1^+^ tumor-infiltrating T cells in three metastatic melanoma specimens were isolated and then stimulated with neoantigens [161]. Neoantigen-specific TCRs were identified from IFN-γ^+^ or IL-2^+^ cells by a single-cell sequencing approach. The frequencies of neoantigen-specific TCRs were estimated to be from 0.58% to 1.79% by this approach. Lastly, T cells specific to neoantigens, melanocyte differentiation antigens or cancer germline antigens were identified in 4.7% to 43.9% of the CD8^+^ TILs isolated from the tumors of four melanoma patients (Section 5.4 and Section 5.5) [154].

Beyond melanoma, the frequencies of neoantigen-specific TCRs ranged from 0% to 1.01% in three colorectal cancer specimens using the same single-cell sequencing approach mentioned previously [161]. In another study, HLA-A*11-tetramers loaded with neoepitopes were used to screen 16 colorectal cancer specimens. Two neoantigen-specific T cell populations were identified from two of these specimens, at 0.11% and 4.38%, respectively [75]. Furthermore, TCRβ deep sequencing was performed on cryopreserved specimens to study the frequencies of the neoantigen-specific TCRs that had identified by neoantigen screening. The frequencies ranged from 0.009% to 1.3% in 10 metastatic GI cancer specimens [162]. As mentioned previously, a report analyzed the neoantigen-specific T cells before and after neoadjuvant therapy for patients with non-small-cell lung cancer (Section 5.5) [159]. According to TCRβ deep sequencing and single-cell sequencing, the frequencies of the neoantigen-specific T cells in the TILs ranged from 0% to 6.3% in seven reported patients. More importantly, pathologic responses were not associated with the frequencies of neoantigen-specific T cells in patients’ tumors or peripheral blood. Taken together, the current evidence strongly supports the role of neoantigen-specific T cells in immunotherapy. However, the detailed mechanisms are still puzzling, including the quantity and quality of neoantigen-specific T cells. The further understanding of the underlying mechanisms will be critical to improve the current immunotherapy approaches.

## 6. Conclusions

**Figure 2:**
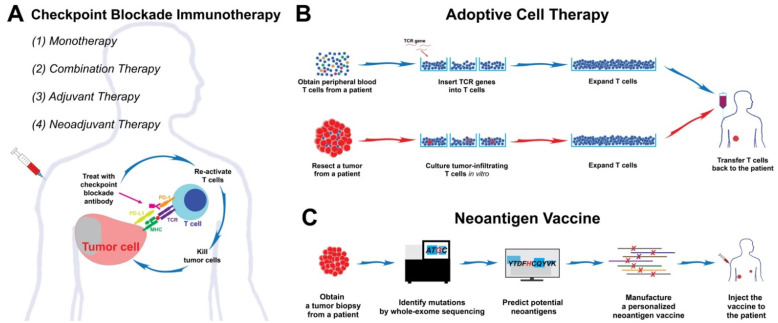
Summary of the current and potentially new immunotherapy options for melanoma. (**A**) The current immunotherapy options are mainly based on the use of checkpoint blockade antibodies. These therapeutic options include monotherapy or combination therapy with other drugs. The preliminary results are made promising by combining the surgical approaches with adjuvant or neoadjuvant immunotherapy. (**B**,**C**) In the near future, adoptive cell therapy (ACT) and neoantigen vaccines may bring new therapeutic options for patients who fail the standard and immunotherapy treatments.

Immunotherapy for advanced melanoma is constantly evolving. The last decade has brought multiple effective and durable treatment options for patients. Promising results in the metastatic setting led to the development of adjuvant and neoadjuvant approaches. As additional immunotherapy agents are developed and the combination and neoadjuvant clinical trials mature, the standard of care for melanoma and the surgical approaches and timing will likely change yet again [8]. In the near future, highly personalized treatments, such as ACT and neoantigen vaccines, may provide new therapeutic options to combat this disease (Figure 2).

## Figures and Tables

**Table 1:** FDA-approved monotherapies used to treat stage III/IV melanoma.

Drug (Manufacturer)	Trial ID	Mechanism of Action	Dosage	Primary Outcome
Interferon (IFN-α-2b) (Merck)	ECOG 1684	Immune stimulator	IFN-α-2b 20 MU/m^2^ intravenously, followed by 10 MU/m^2^ subcutaneously	Median OS: 3.8 years (IFN-α-2b) vs. 2.8 years (observation)
Aldesleukin (Clinigen)		T-cell stimulator	600,000 or 720,000 IU/kg	Median OS: 11.4 months
Ipilimumab (Bristol-Myers Squibb)	CA 184-002 (NCT00094653)	CTLA-4 checkpoint inhibitor	Ipi 3 mg/kg + gp100	Median OS: 10.0 months (Ipi + gp100) vs. 6.4 months (gp100 alone)
	CA 184-169 (NCT01515189)		Ipi 10 mg/kg vs. Ipi 3 mg/kg	Median OS: 15.7 months (10 m/kg) vs. 11.5 months (3 mg/kg)
Pembrolizumab (Merck)	KEYNOTE-002 (NCT01704287)	PD-1 checkpoint inhibitor	Pembro 2 mg/kg vs. 10 mg/kg vs. chemo	6 month PFS: 34% (Pembro 2 mg/kg), 38% (Pembro 10 mg/kg), 16% (chemo)
	KEYNOTE-006 (NCT01866319)		Pembro 10 mg/kg vs. Ipi 3 mg/kg	Median OS: 32.7 months (Pembro) vs. 15.9 months (Ipi)
Nivolumab (Bristol-Myers Squibb)	Checkmate-066 (NCT01721772)	PD-1 checkpoint inhibitor	Nivo 3 mg/kg vs dacarbazine 1000 mg/m^2^	3 yr OS 51.2% (Nivo) vs. 21.6% (Dab)
	Checkmate-067 (NCT01844505)		Nivo 3 mg/kg vs. Ipi 3 mg/kg	5 yr OS 44% (Nivo) vs. 26% (Ipi)
Talimogene laherparepve (T-VEC) (Amgen)	OPTiM (NCT00769704)	Oncolytic virus	Up to 4 mL of 10⁸ pfu/mL per intratumoral injection vs. GM-CSF 125 μg/m^2^	Median OS: 23.3 months (T-VEC) vs. 18.9 months (GM-CSF)
Vemurafenib (Genentech)	BRIM-3 (NCT01006980)	BRAF inhibitor	Oral Vem 960 mg vs. dacarbazine 1000 mg/m^2^	Median OS: 13.6 months (Vem) vs. 9.7 months (dacarbazine)
Dabrafenib (Novartis)	BREAK-2 (NCT01153763)	BRAF inhibitor	Oral Dab 150 mg	OS at 3, 4, 5 years: 30%, 23%, 20%
	BREAK-3 (NCT01227889)			OS at 3, 4, 5 years: 31%, 27%, 24%

Abbreviations: Ipi—ipilimumab; Pembro—pembrolizumab; Nivo—nivolumab; T-VEC—talimogene laherparepvec; Vem—vemurafenib; Dab—dabrafenib; PFS—progression free survival; OS—overall survival.

**Table 2:** FDA approved combination therapies used to treat stage III/IV melanoma.

Drug (Manufacturer)	Trial ID	Mechanism of Action	Dosage	Primary Outcome
Dabrafenib + trametinib (Novartis)	COMBI-d (NCT01584648)COMBI-v (NCT01597908)	BRAF inhibitor + MEK inhibitor	Dab 150 mg + Tram 2 mg	OS at 2 & 3 years: 52%, 44%
Vemurafenib + cobimetinib (Genentech)	CO-BRIM (NCT01689519)	BRAF inhibitor + MEK inhibitor	Vem 960 mg + Cobi 60 mg	Median PFS: 9.9 months
Encorafenib + binimetinib (Pfizer)	COLUMBUS (NCT01909453)	BRAF inhibitor + MEK inhibitor	450 mg encorafenib + 45 mg binimetinib	Median PFS: 14.9 months
Atezolizumab + vemurafenib + cobimetinib (Genentech)	IMspire 150 (NCT02908672)	PD-L1 checkpoint inhibitor + BRAF inhibitor + MEK inhibitor	cycle 1: Vem 960 mg for 21 days + Cob 60 mg, followed by Vem 720 mg cycle 2: atezolizumab 840 mg, Vem 720 mg, Cob 60 mg	Median PFS: 15.1 months
Nivolumab + ipilimumab (Bristol-Myers Squibb)	Checkmate-067 (NCT01844505) Checkmate-069 (NCT01927419)	PD-1 checkpoint inhibitor + CTLA-4 checkpoint inhibitor	Nivo 1 mg/kg + Ipi 3 mg/kg, followed by Nivo 3 mg/kg	ORR: 58%

Abbreviations: CI—confidence interval; HR—hazard ratio; Dab—dabrafenib; Tram—trametinib; Vem—vemurafenib; Cob—cobimetinib; Nivo—nivolumab; Ipi—ipilimumab; Pembro—pembrolizumab; PFS—progression free survival; ORR—overall response rate.
